# The North Pole at Last!

**Published:** 1909-10

**Authors:** 


### The North Pole at Last!

DR. FREDERICK A. COOK startled the world by his announcement from Lerwick, Shetland Islands, published in the newspapers of September 2, that he had visited the North Pole, and was returning to Copenhagen by the steamer Hans Egede. The despatch further states that he reached the Pole April 21, 1908, and his further advice informed the public that he remained at the Pole for two days. He soon landed in Copenhagen, where he was received with demonstrations of enthusiasm on all sides. It is a matter of pride that the first white man to visit the Pole should be a member of the medical profession.

Dr. Cook graduated from the Medical Department of the University of the City of New York in the class of 1891.

He was born in Callicoon, Sullivan County, N. Y., on June 10, 1865. He was graduated from New York University and from the College of Physicians and Surgeons of Columbia University in 1890. Two years later he married Miss Mary Hunt, of Brooklyn.

Dr. Cook went north with the Peary Arctic expedition of 1891-'92 as its surgeon, commanded the Zeta, a yacht, on a northern cruise in 1893, and in 1894 organised and led a scientific expedition to the Greenland coast, which was in numbers one of the largest of northern ventures. Fifty students and investigators sailed with him in the steamship Miranda, of the Red Cross Line, a ship of 1,100 tons.

The cruise of the Belgica was one of the most important of Antartic expeditions. She was a small bark, which was fitted out partly by the Belgian government and partly by private subscription, to explore the seas below the Antartic Circle. She sailed from Belgium in August of 1897, and at Rio de Janeiro was joined by Dr. Cook in the capacity of surgeon-in-chief and anthropologist.

The little ship was caught in the heavy ice pack and carried with the drift for 2,000 miles. From May, 1898, to February, 1899, the ship was frozen in the center of an ice field about two miles square. She made Punta Arenas on March 14, 1899. The most important results of the expedition were contained in the magnetic observations, covering an entire year, which indicated that the Southern magnetic pole was 200 miles farther east than the observations of Sir James Ross had seemed to show.

In 1901, Dr. Cook returned to the Arctic seas in the expedition of the Eric, the Peary expedition auxiliary.

Other medical men have made attempts to visit the North Pole before Dr. Cook's time, one of the most noted expeditions being that of Dr. Elisha Kent Kane. In this he was associated with Dr. Hayes, who later, himself, made an attempt to reach the goal. Dr. Richardson in 1826, and Dr. John Rae in 1845, are the names of other physicians who have made unsuccessful attempts to visit the Pole, but have, nevertheless, contributed much to our knowledge of geography with the Arctic Circle. The lesson to be drawn from these various attempts is, that a medical training is a valuable asset for those who attempt to discover the unknown.

Since writing the foregoing, Dr. Cook has returned to America, and has been received with great honor, even with a loud acclaim of joy, such a greeting as befits his heroic achievement.

